# Long distance movement of an Arabidopsis Translationally Controlled Tumor Protein (*AtTCTP2*) mRNA and protein in tobacco

**DOI:** 10.3389/fpls.2014.00705

**Published:** 2014-12-17

**Authors:** Roberto Toscano-Morales, Beatriz Xoconostle-Cázares, Angélica C. Martínez-Navarro, Roberto Ruiz-Medrano

**Affiliations:** Department of Biotechnology and Bioengineering, Centro de Investigación y de Estudios Avanzados del Instituto Politécnico NacionalMexico, Mexico

**Keywords:** TCTP (Translationally Controlled Tumor Protein), long-distance movement, aerial roots, adventitious roots, non-cell autonomous protein

## Abstract

Translationally Controlled Tumor Protein (TCTP) is an almost ubiquitous protein found in eukaryotes, fundamental for the regulation of development and general growth. The multiple functions of TCTP have been inferred from its involvement in several cell pathways, but the specific function of TCTP is still not known in detail. On the other hand, TCTP seems to respond to a plethora of external signals, and appears to be regulated at the transcriptional and/or translational levels by mechanisms yet to be determined. In the present work, we analyzed the capacity of *AtTCTP2* gene products (mRNA and protein) to translocate long distance through tobacco heterografts (transgenic/WT and WT/transgenic). The results indicate that both *AtTCTP2* mRNA and protein are capable of moving long distance in both directions (stock-scion and scion-stock) with a tendency for movement from source to sink tissue (stock to scion). Interestingly, aerial roots emerged only in heterografts where the protein was detected in both stock and scion, suggesting a correlation between the presence of AtTCTP2 and aerial root appearance. More detailed analysis showed that these aerial roots harbored the transgene and expressed both transcript and protein. In addition, the protein localization pattern in transgenic aerial and primary roots was basically the same, indicating specific nuclear destination in roots, but also in leaves. These findings provide an approach to understand the role of long-distance movement in the function of plant TCTPs, supporting the notion that some of these act in a non-cell autonomous manner, as the human counterpart, the Histamine Releasing Factor (HRF).

## Introduction

Translationally Controlled Tumor Protein (TCTP) is a highly conserved protein family found in almost all eukaryotes, which has been associated to the regulation of important biological processes, including cell proliferation and differentiation in animals (Bommer and Thiele, 2004; Chen et al., 2007; Hsu et al., 2007) as well as in plants (Berkowitz et al., 2008; Brioudes et al., 2010). TCTP has been considered a multifunctional protein due to its capacity to interact with diverse targets, such as factors involved in apoptosis, protein synthesis, cell repair, cytoskeletal arrange, and general metabolism (Bommer, 2012). However, the specific role of TCTP in plants and eukaryotes in general, is still a theme of debate. In this regard, in spite of the evident relevance of TCTP in growth and developmental regulation for most eukaryotes, the functions that this protein exerts seem to be cell-, tissue-, and/or developmental stage-dependent, making it difficult to determine the specific mechanisms through which TCTP acts.

It has been proposed that TCTP is related to small GTP-binding proteins, presumably with a role as a guanine nucleotide exchange factor (GEF) to activate Ras small GTPases (Thaw et al., 2001; Hsu et al., 2007), which regulate cell proliferation, cytoskeletal dynamics/morphology, membrane trafficking, and are part of the mTOR pathway, although this last has been disputed (Wang et al., 2008). Indeed, TCTP may have a guanine nucleotide dissociation activity (Cans et al., 2003), specifically acting during protein synthesis through the stabilization of the GDP form of the elongation factor eEF1A and impairing the GDP exchange reaction promoted by eEF1B (GEF; Hsu et al., 2007). Moreover, in *Drosophila*, alterations in some of the putative G-protein binding sites resulted in TCTP inactivation resembling the proliferation and growth defects seen in TCTP silencing (Hsu et al., 2007).

In plants, the role of TCTP may be related to growth and developmental regulation (Woo and Hawes, 1997; Berkowitz et al., 2008; Brioudes et al., 2010; Nakkaew et al., 2010), as in most eukaryotes. In fact, these functions seem to be highly conserved across kingdoms given that Arabidopsis TCTP (AtTCTP1; At3g16640) is capable of complementing a *Drosophila* TCTP mutant and vice versa (Brioudes et al., 2010). However, plant TCTPs have been also associated to several other functions such as response to water deficit (Kim et al., 2012), male gametophyte maturation (Berkowitz et al., 2008), defense response (Jones et al., 2006; Yan et al., 2009; Cao et al., 2010), endosperm development (Qin et al., 2011), storage root formation (de Souza et al., 2004), fruit ripening (Lopez and Franco, 2006), and photoperiodism and flowering (Sage-Ono et al., 1998), given that the corresponding genes are induced during the aforementioned conditions.

Several of the previously mentioned studies on TCTP have shown that this protein is ubiquitous and is highly expressed in most tissues across several plant species, but also that it might be regulated (posttranscriptionally and translationally) in response to a wide range of extracellular stimuli in multiple seemingly unrelated cellular processes (Bommer, 2012). The few studies carried out in different species indicate high levels of both mRNA and protein relative to constitutive genes. There is little information regarding plant TCTP posttranscriptional or translational regulation. Overall, in Arabidopsis, the levels of *AtTCTP1* transcript and protein are correlated (Berkowitz et al., 2008), although low protein levels have been observed in inflorescence stem that did not correlate to the abundance of its mRNA, suggesting posttranscriptional or translational regulation (Brioudes et al., 2010). Similar results have been found for the *Cucurbita maxima* TCTP (CmTCTP); while elevated levels of the transcript are found in the shoot apex almost no protein is detected in this tissue (Hinojosa-Moya et al., 2013). Transcript and protein may also be regulated at the level of its cell-specific localization and intercellular movement; indeed, in some species TCTP transcript and protein are found in the phloem translocation stream, suggesting its long-distance movement (Lin et al., 2009; Rodríguez-Medina et al., 2011; Hinojosa-Moya et al., 2013). TCTP appears to interact with the phloem RNA-binding protein CmPP16 (Aoki et al., 2005). More recently, the movement of the Arabidopsis TCTP mRNA to dodder (*Cuscuta reflexa*) as well as of several other transcripts has been reported (Kim et al., 2014). All this implies a possible non-cell autonomous function, in a manner analogous to the Histamine Releasing Factor (HRF) from diverse animal taxa, which is a member of the TCTP superfamily.

Recent studies from our group have suggested a division of labor among TCTPs between plants that harbor more than one gene (Gutiérrez-Galeano et al., 2014). This is the case of Arabidopsis, which harbors two TCTP genes; one of them, *AtTCTP1* (At3g16640), has a role in control of mitotic growth (Berkowitz et al., 2008; Brioudes et al., 2010); the other, *AtTCTP2* (At3g05540), has been considered a pseudogene. Nonetheless, we have found that this gene is expressed and may be functional, since it can induce tobacco regeneration when harbored by *Agrobacterium rhizogenes*, in a manner analogous to *CmTCTP*, and that the mRNA was detected, albeit at much lower levels than the *AtTCTP1* mRNA; also that the promoter is functional (Hinojosa-Moya et al., 2013; Toscano-Morales et al., submitted). In the present report we took advantage of the capacity of AtTCTP2 to induce whole plant regeneration in tobacco to obtain transgenic tobacco plants harboring a *35S::AtTCTP2-GFP* construct. Wild type plants were grafted onto these transgenic plants, to test for AtTCTP2 long-distance movement from transgenic stocks to wild-type scions. It was found that *AtTCTP2* mRNA, as well as its protein, are capable of long distance movement, underlying the possible non-cell autonomous function of AtTCTP2.

## Materials and methods

### Assembly of *AtTCTP2-GFP* overexpression construct

PCR amplification and cloning of the *AtTCTP2* ORF into the pCR8/GW/TOPO vector (Invitrogen, Carlsbad CA) was carried out previously (Toscano-Morales et al., submitted). Briefly, the *AtTCTP2* ORF was recombined into the pB7FWG2 Gateway binary vector (Plant Genetic Systems, Ghent, Belgium). The resulting construct was then introduced by electroporation into *A. rhizogenes* strain K599.

### Explant transformation/regeneration of transgenic tobacco plants

Transformation of tobacco leaf explants was carried out by the puncture method described by Hinojosa-Moya et al. (2013). Basically, cultures containing *A. rhizogenes* K599 harboring the recombinant plasmids were grown on selective liquid media, concentrated, and resuspended in fresh media. Young tobacco leaves (2–3 cm diameter) were sterilized using ethanol 70% (1 min) followed by soaked in 10% sodium hypochlorite for 30 min and several washes with sterile water. Then, these leaves were inoculated with the previously prepared bacterial suspension employing a sterile insulin syringe. After transformation the explants were placed on MS medium with no hormone supplementation [1.0 MS salts, 2% sucrose, and 0.4% agar (Gelrite)] and finally transferred to controlled environment growth chamber under long-day conditions (16 h light/8 h dark) during 30 days. The regenerated plantlets were transferred to sterile-soil, watered and kept in transparent plastic bags to maintain their turgor and low CO_2_ concentrations. One week later, plastic bags were removed and plants kept under greenhouse conditions until reaching maturity.

### Transgenic plant selection

F1 seeds were harvested and sowed on soil, where these were subject to herbicide (Ammonium glufosinate-FINALE, Bayer, Germany) selection to isolate the transgenic offspring (segregation analysis were not performed). Total DNA was extracted from all candidate plants (including wild type) and each one of these was used as template for transgene PCR detection, employing GFP primers (forward: 5′-ATGGTGAGCAAGGGCGAGGAGCTG-3′; reverse: 5′-CCTTGTACAGCTCGTCCATGC-3′). PCR positive plants were then isolated in individual pots and grown 1–2 weeks under greenhouse conditions.

### Tobacco grafting assays

Grafting assays were conducted using a combination of the grafting methods described by Palauqui et al. (1997) and Imlau et al. (1999). Essentially, the stock is decapitated 20–25 cm above the soil, and the outermost cortex of the stock stem is cut longitudinally to produce a cortex flap; after this the terminal apex of the scion (carrying 2–3 leaves of 0.5–1 cm) is excised, chamfered and attached to the stock in the gap between the flap and the stem, to finally be wrapped with parafilm. To avoid desiccation and turgor loss, grafted plants were maintained under transparent plastic bags for 10 days under controlled conditions in a growth chamber. Then, plastic bags were removed and plants were kept for another 5–7 days under these conditions. Finally, grafted plants were transferred to a greenhouse; 2–3 weeks later, newly developed sink leaves near the scion apex (<1 cm) and fully expanded source leaves in the stock (>2 cm) were harvested, frozen with liquid nitrogen and stored at −80°C for further use.

### mRNA RT-PCR detection

Total RNA was extracted from each sample, with an average weight of 15–20 mg per sample, using the RNAeasy Kit (QIAgen, Hilden, Germany). Total RNA concentrations from samples were normalized, and used as templates to perform one-step RT-PCR reactions for each sample employing a commercial RT-PCR system (KAPA FAST Universal One-Step q-RT PCR Kit) in order to detect *AtTCTP2-GFP* and *18S* (as control) mRNAs using specific primers [GFP (see above, transgenic plant selection) and 18S (direct: 5′-GCCCGGGTAATCTTTGAAATTTCAT-3′; reverse: 5′-GTGTGTACAAAGGGCAGGGACGTA-3′)]. The conditions of the one step RT-PCR were as follows: (1) 42°C–10 min, (2) 95°C–5 min, and (3) 95°C–5 s/62°C–30 s/72°C–15 s (40 cycles). Finally, amplicons were visualized on a 1.2% agarose gel. The amplicons obtained are 720 bp for *AtTCTP2-GFP* and 152 bp for *18S*.

### Quantitative RT-PCR

GFP, AtTCTP2, BAR, NtTCTP, and 18S RNA levels were determined as follows: total RNA was extracted for each graft sample (stock or scion) and used for one-step RT-PCR (100 ng in a 10 μL reaction). A commercial system was used according to the manufacturer′s recommendations (KAPA SYBR FAST Universal One-Step qRT-PCR Kit). Specific primers for GFP, 18S (shown previously in this section), AtTCTP2 (direct: 5′-ATGTTGGTCTACCAGGATATTCTTACA-3′; reverse: 5′-GCACTTGATCTCTTTCAAGCCGTAGGC-3′), and NtTCTP (direct: 5′-GGAAGTGGGTTGTTCAGGGAGCTGTTGATG-3′; reverse: 5′-TGTTCTTAAAAACTTCTTCCTGCTCTGCGCCT-3′) were used. The Real Time qRT-PCR reactions were incubated in a Rotor Gene 3000 apparatus (Corbett Research, Australia) using the following PCR conditions: 5 min at 42°C for reverse transcription followed by 3 min at 95°C with 40 cycles of denaturation (95°C for 3 s), annealing (60°C for 20 s) and extension (72°C for 3 s). To verify that no additional products were amplified in the reaction, a dissociation curve was generated through progressive sample heating (60–95°C). The Ct value for each product was determined by triplicate in each treatment. 18S rRNA mRNA was used to normalize gene expression. Relative quantification for transcript accumulation was performed according to the comparative ΔΔ CT method (Livak and Schmittgen, 2001) relating fold changes in GFP, AtTCTP2, between transgenic samples and its WT counterpart (either stocks or scions).

### Total protein extraction and histochemical detection

Western blot assay was performed as described by Hinojosa-Moya et al. (2013). In resume, total protein was extracted by grinding plant tissue in liquid nitrogen, homogenized in protein extraction buffer, and centrifuged at 13,000 g for 2 min. Supernatants were recovered and dissolved in SDS-PAGE sample buffer. Protein concentration was determined using a spectrophotometer (NanoDrop™ 1000; Thermo Scientific, Waltham, MA) and all samples homogenized to the same concentration followed by 12% SDS-PAGE. Total proteins were transferred for 1 h at 100 V to polyvinylidene difluoride (PVDF) membranes (Whatman), blocked for 2 h in blocking solution (PBS 1X, 5% non-fat milk, and 0.1% Tween 20) followed by rinsing with PBS 1X, and incubated overnight at 4°C with the polyclonal GFP antibody (diluted 1:2000 in 1X PBS, 5% non-fat milk, and 0.1% Tween 20). Membranes were washed (1X PBS, 0.1% Tween 20) 5 times and incubated with horseradish peroxidase (HRP)-conjugated goat anti-rabbit IgG (Santa Cruz Biotechnology, CA) at 1:5000 in PBS 1X containing 2.5% skim milk during 2 h. Finally, after several washes, the signal was detected with HRP color development reagent (Amersham Prime Western Blotting Kit Reaction-ECL™-GE Healthcare™) and revealed in an Amersham Hyperfilm (ECL™-GE Healthcare™) film.

### Confocal fluorescence microscopy for *AtTCTP2-GFP* detection

Samples from stocks and scions were analyzed on a Leica confocal laser scanning microscope (model TC-SP5/MO-TANDEM) using a krypton/argon with a laser excitation fluorescence/emission of 488/525 nm for green fluorescence and 580 nm/665 nm emission. All images were recorded and analyzed with Leica Las AF software, followed by processing using Photoshop 8.0 software (Adobe) as described (Xoconostle-Cázares et al., 1999).

### Phenotypic visualization of adventitious roots

Parafilm-junction was removed from grafted plants after 30 days, after which adventitious root appearance was visualized using a Sony Cybershot DSC-W300 digital camera (13.6 megapixels) harboring a 3× (35–105 mm) Carl Zeiss Vario-Tessar lens f/2.8–5.5.

## Results

### *AtTCTP2-GFP* mRNA and protein are detected in wild type tobacco grafted onto transgenic tobacco

**Figure 1:**
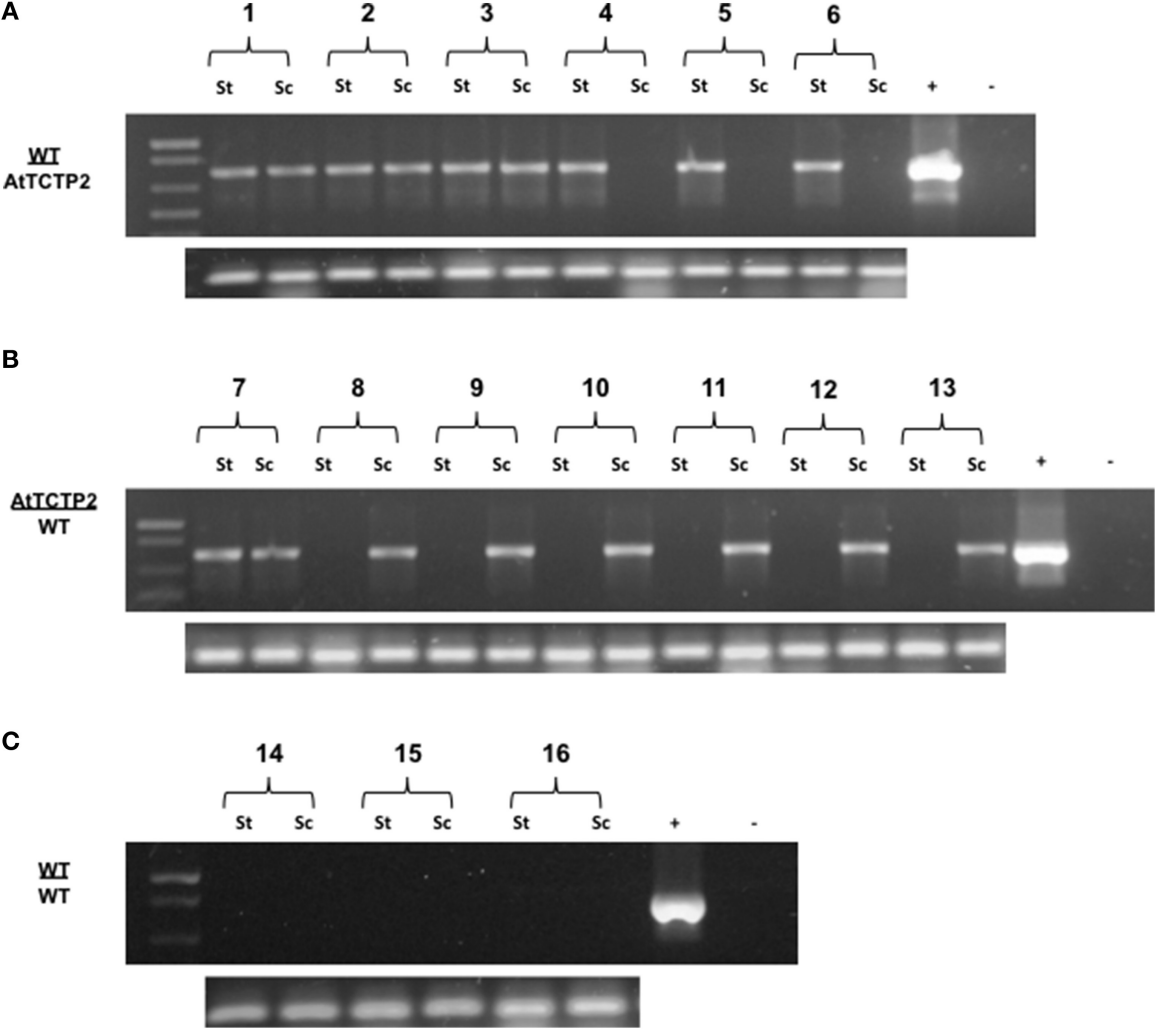
**Detection of AtTCTP2-GFP mRNA long-distance movement in tobacco grafts**. Total RNA samples from stocks and scions (transgenic or WT) were extracted and used as templates to perform RT-PCR detection of the transcript AtTCTP2-GFP. For further propose we denote grafts as scion (apex) genotype/rootstock genotype. **(A)** In WT/AtTCTP2 grafts the mRNA of AtTCTP2-GFP was detected in half of the wt scions (see Table 1), while in **(B)** AtTCTP2/WT grafts the transcript was detected in one wt stock from the seven grafts performed (see Table 1). **(C)** Homografts WT/WT were carried out as controls, and no signal was detected. RT-PCR detection of 18S was performed as control for RNA quality in all samples (below **A**–**C**).

Transgenic tobacco plants harboring the overexpression construct *35S:AtTCTP2-GFP-TNOS* were obtained using the regeneration protocol described by Hinojosa-Moya et al. (2013). Taking advantage of this, the progeny of these regenerated plants was selected for herbicide resistance and tested for presence of trangene by PCR. Transgenic and WT plants of similar size and developmental stage were used to perform reciprocal heterografting experiments as well as WT homografts. Total RNA was extracted from young and mature leaves in all grafting experiments; these were used as templates to perform the RT-PCR detection of the *AtTCTP2-GFP* transcript to test for long-distance movement. Signal corresponding to GFP was detected in three out of six WT scions grafted onto transgenic stocks (Table 1), indicating long-distance movement of this mRNA (Figure 1A). Interestingly, in the case of the reciprocal heterograft, this signal was identified in one of seven grafts (Table 1, Figure 1B) suggesting directionality of the movement of this mRNA, likely from stock to scion (AtTCTP2-GFP/WT). The control homografts (WT/WT) did not show any transgenic signal (Figure 1C); *18S* RNA was detected in all the samples at similar levels.

**Table 1:** **AtTCTP2-GFP long-distance movement as mRNA and protein in tobacco graftings**.

**Grafting type**	**mRNA detection (RT-PCR)**	**Protein detection (confocal microscopy)**	**Advenitious root emergence**
AtTCTP2-GFP/WT	3/6	6/6	6/6
WT/AtTCTP2-GFP	1/7	4/7	4/7
WT/WT	0/3	0/3	0/3

**Figure 2:**
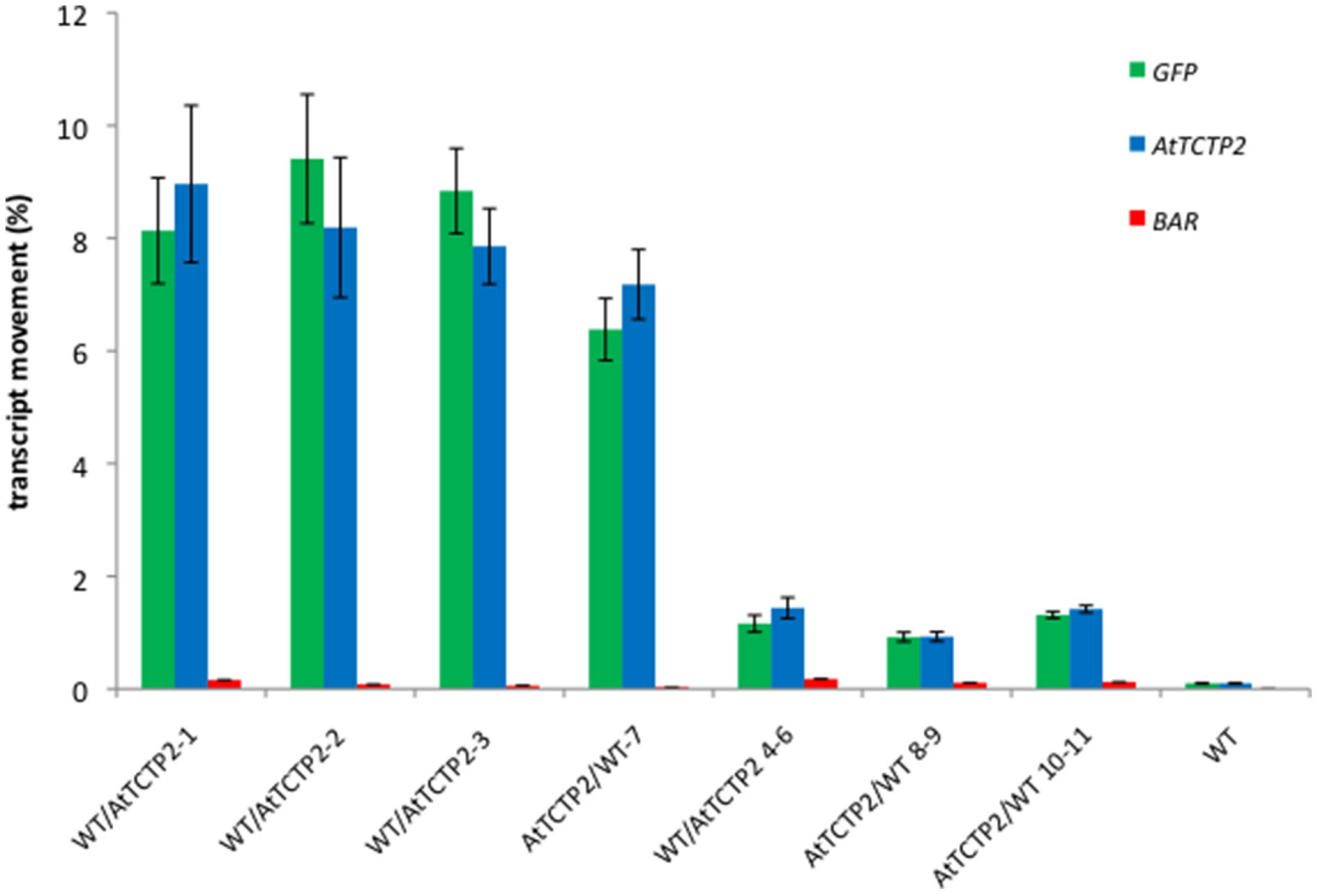
**Quantification of AtTCTP2-GFP mRNA long distance movement suggests preference for directional movement from rootstock to scion**. Total RNA was extracted from transgenic or WT rootstocks and scions and homogenized to a given concentration (100 ng/μl) to be used as templates to perform Real Time qRT-PCR. GFP (green) and AtTCTP2 (blue) were quantified in all cases, using BAR as a negative control for mRNA movement. mRNA movement percentages for GFP, AtTCTP2 and BAR from rootstock to scion (WT/AtTCTP2-) and vice versa (AtTCTP2/WT) were determined by relating fold changes between transgenic and wt components on the graft after normalizing against 18S as reference gene. Three technical replicates were performed in each case, given as means ± SE.

The movement of AtTCTP2-GFP mRNA from transgenic stocks to WT scions, and from transgenic scions to WT rootstocks was determined by quantitative RT-PCR. The results indicate that the percentage of transcript that moved across the graft union, obtained as the ratio of *AtTCTP2-GFP* mRNA present in the non-transgenic scion, and that in the transgenic stock, is between 7 and 9% (Figure 2). Interestingly, *AtTCTP-GFP* mRNA was also detected moving rootward from transgenic scion to non-transgenic WT stock, albeit at lower efficiencies (between 1 and 1.5%).

To discard that only *AtTCTP2-GFP* transcript is capable of moving long-distance, the presence of *npt2* mRNA was analyzed in WT/*AtTCTP2-GFP* and *AtTCTP2-GFP/*WT heterografted plants, as well as in WT controls. Quantitative RT-PCR was carried out for this end. As shown in Figure 2, no *npt2* mRNA was detected in non-transgenic scions or stocks grafted to *AtTCTP2-GFP* expressing plants. Thus, only *AtTCTP2-GFP* mRNA is transported long-distance.

### *AtTCTP2GFP* protein also moves through a graft union

**Figure 3:**
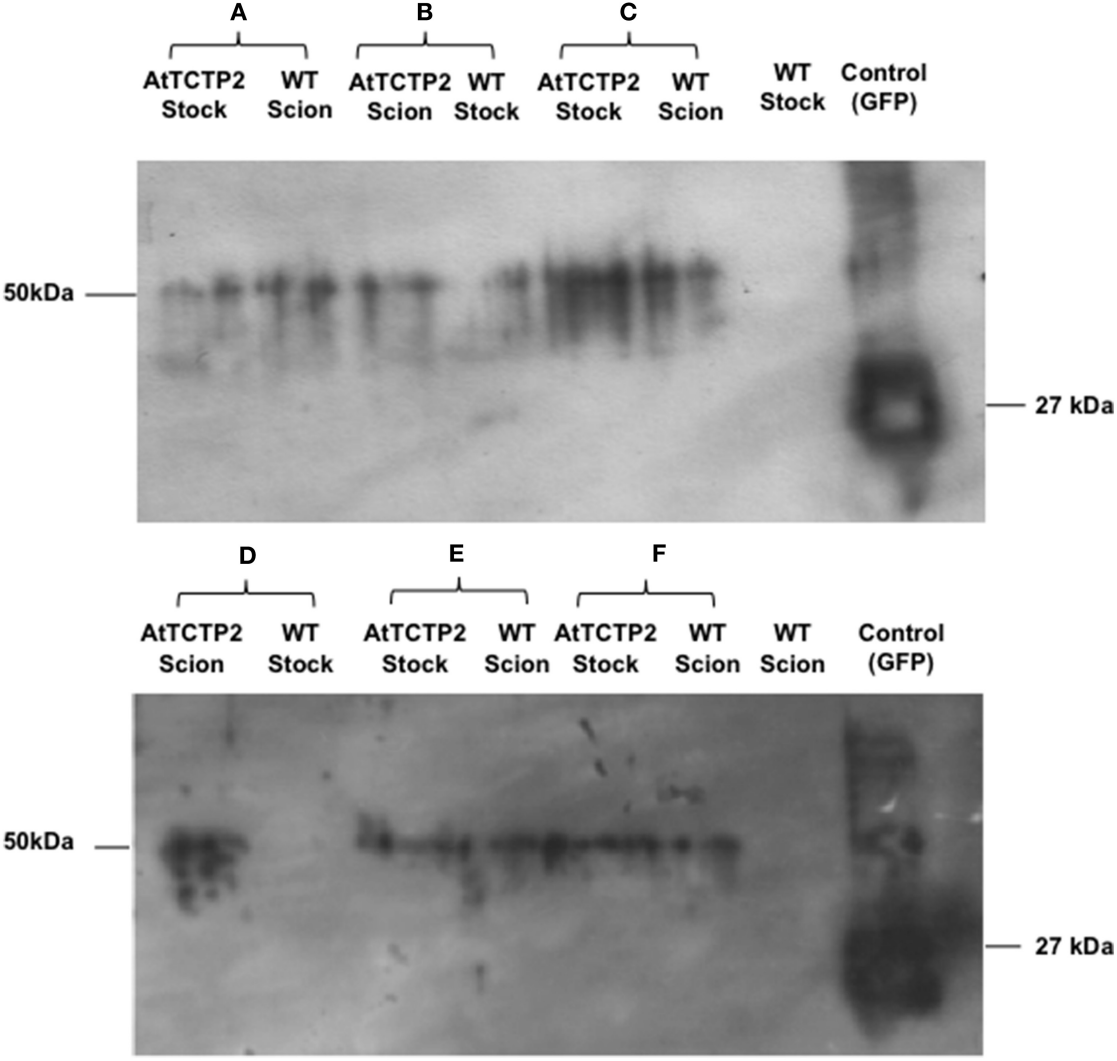
**AtTCTP2-GFP protein moves long distance in tobacco grafts**. Inmunodetection was performed to detect GFP fused to AtTCTP2 from scions and stocks for each graft tested. Panels **(A,C,E,F)** are examples of the fusion protein long-distance movement (AtTCTP2-GFP = 50 kDa) from rootstock to scion. Panel **(B)** is the unique example of AtTCTP2-GFP long distance transport from scion to rootstock. Panel **(D)** is a representative example of lack of AtTCTP2-GFP long-distance movement from scion to rootstock.

The previous results could not discriminate whether the AtTCTP2-GFP transcript, the protein, or both, were able to move across the graft union. In order to answer this question, the presence of the fusion protein was determined by Western blot analysis of total proteins from scions and stocks of transgenic plants. Independent scions and stocks were used in which AtTCTP2 mRNA movement was observed. In all cases, the fusion protein was detected in both WT scion grafted onto a transgenic stock; in the case of WT stock onto which a transgenic scion had been grafted, one out of two AtTCTP2 could be detected in the former (Figure 3). These results indicate that AtTCTP2-GFP moves long distance as both mRNA and protein, and that such movement is not always in the direction from source to sink.

**Figure 4:**
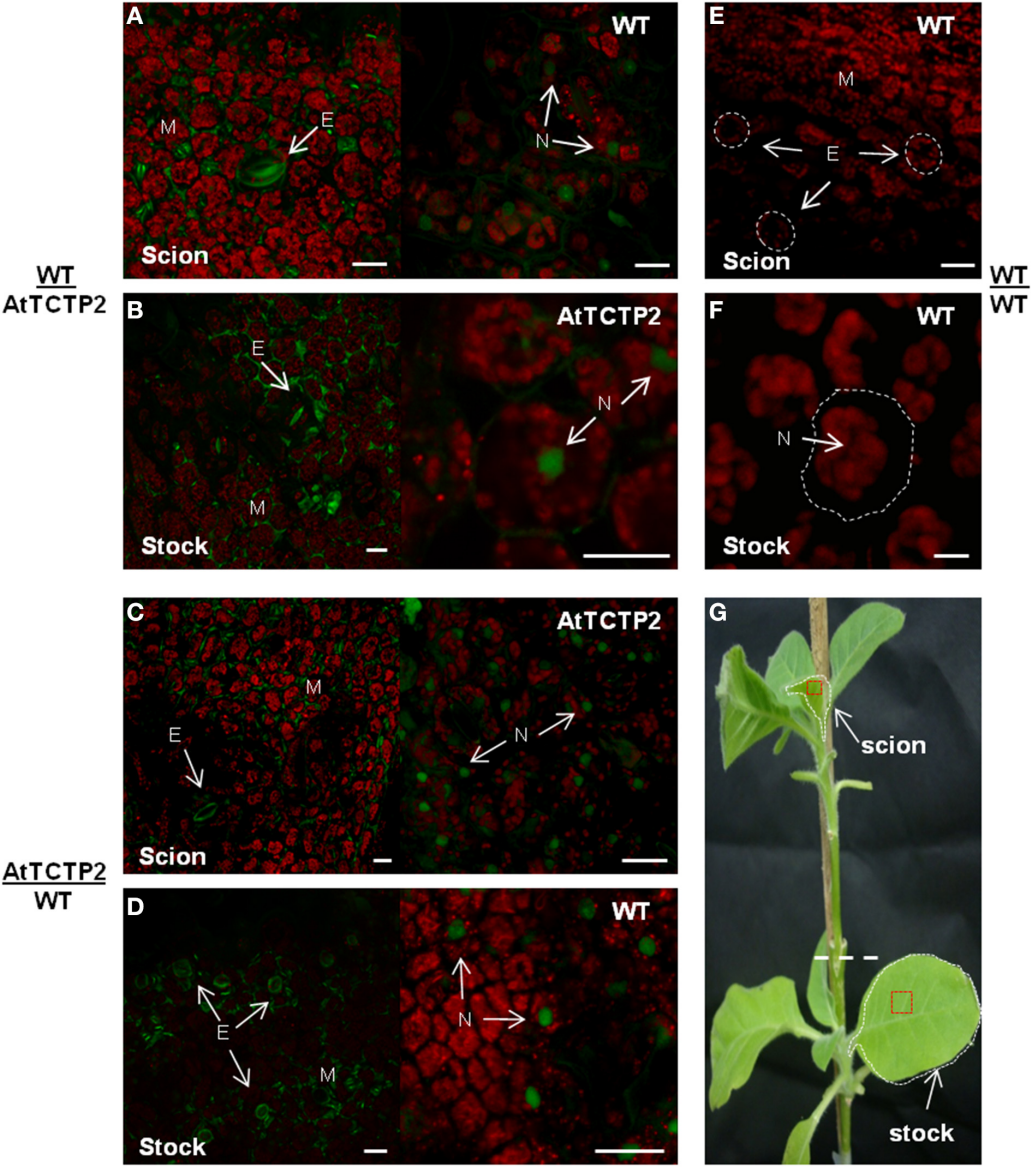
**AtTCTP2-GFP accumulation sites are conserved**. Laser confocal microscopy was performed to detect the fluorescence associated to GFP fused to AtTCTP2 both in young-leaves near apical meristems (from scions) and in source well developed leaves (from stocks) for each graft tested. In WT/AtTCTP2 grafts the fusion protein AtTCTP2-GFP was identified in both **(A)** stock and **(B)** scion in all grafts (see Table 1) presenting the same localization pattern characterized by signal detection in stomata (S) and nuclei (N), besides mesophyll (M). **(C,D)** In AtTCTP2/WT grafts the fusion protein showed the same localization pattern previously described, but the signal was only observed in four wt stocks of the seven grafts performed (see Table 1). WT/WT homografts were used as controls, where no fluorescence signal was detected in **(E)** scion or **(F)** stock. **(G)** Visual representation of grafts separated by a white dashed line, the selected tissue for RT-PCR is delimited by white dashed lines and for fluorescence confocal microscopy in red squares. Size bars: 25 μm.

To confirm the long-distance movement of AtTCTP2 protein, as well as the localization pattern of the fluorescent fusion protein, confocal microscopy was used to detect GFP-associated fluorescence and thus AtTCTP2. Tissue samples were collected from the same leaves used for RT-PCR (marked in red in Figure 4G), and prepared for confocal analysis. GFP fluorescence was detected in mesophyll, stomata and nuclei in leaf tissue from transgenic stocks or scions (Figures 4A,D), which is the same accumulation pattern of AtTCTP2 in Arabidopsis (Toscano-Morales et al., submitted). GFP signal was also identified in all WT scions and in four out of seven stocks (Figures 4B,E; Table 1) showing a highly similar pattern of localization. No signal was detected in WT/WT controls either in stock or scion (Figures 4C,F). These observations suggest the AtTCTP2-GFP fusion protein moves long distance in both directions, but preferentially from stock to scion (Table 1). Interestingly, AtTCTP2-GFP protein was detected in all WT scions, even in those samples negative by RT-PCR (Table 1). This also suggests that the fluorescence signal detected could not only be attributed to the translation of this mRNA once it moved long-distance but to the actual movement of AtTCTP2 as protein. Overall, both RT-PCR and fluorescence detection of AtTCTP2-GFP indicate its capacity for long-distance movement as protein and/or as transcript.

### Emergence of aerial (adventitious) roots at the graft interface correlates with *AtTCTP2* protein movement

**Figure 5:**
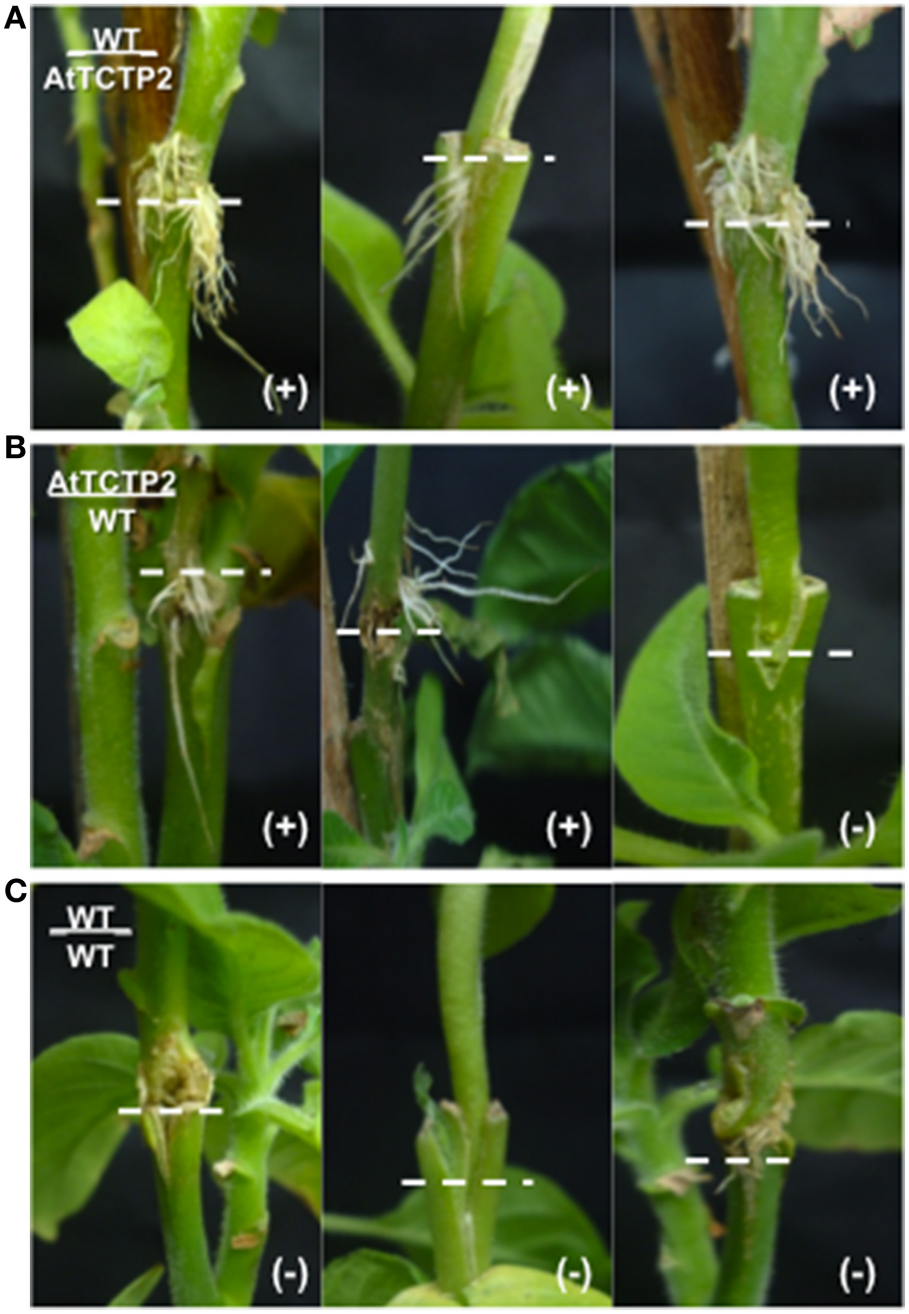
**Emergence of adventitious roots associated to long-distance movement of AtTCTP2**. Representative images were taken previous tissue collection for RT-PCR and confocal analysis (30 days). **(A)** WT/AtTCTP2 grafts presented aerial roots in all samples, which is consistent with the protein detection in the same graft samples (see Table 1). (**B**: left and middle) AtTCTP2/WT grafts produced aerial roots in four out of seven trials, correlating specifically to the presence of AtTCTP2-GFP protein in the same graft samples (see Table 1 and Figure 2). (**B**: right) Hence, in the samples where no aerial roots were present no protein was observed. **(C)** The latter is consistent to the WT/WT graft controls.

Adventitious roots are normally found in some plant species and have a role in adaptation to stress or nutrient deficiency (Drew et al., 1979), as well as for vegetative propagation of some tree species (Naiman and Décamps, 1997). The emergence of aerial roots in the adventitious region in the graft interface was observed in several of the grafting experiments performed (Figure 5). Indeed, such adventitious roots were observed in the sites adjacent to the graft union in all AtTCTP2/WT grafts (Figure 5A), and in some of the WT/AtTCTP2 grafts (Figure 5B) but not in the control (Figure 5C). Interestingly, the appearance of these adventitious roots was specifically associated to the grafts where GFP-associated fluorescence (and hence AtTCTP2) was detected in WT scion or stock, and thus, in which long-distance transport of the protein occurred (Table 1); interestingly, in some cases fluorescence was observed, even though *AtTCTP2* mRNA was detected. This suggests that the emergence of these aerial roots is linked to the movement of the protein rather than to movement of the transcript. No GFP-associated fluorescence was observed in equivalent tissues from WT homografts.

### *AtTCTP2-GFP* nuclear localization in primary and adventitious roots suggests directionality of protein movement across the graft union

Adventitious aerial roots were analyzed by final point PCR and RT-qPCR to determine the presence of the transgene and its expression levels. All aerial roots were transgenic (harboring the *35S::AtTCTP2-GFP* construct) and positive for the *AtTCTP2-GFP* transcript (data not shown). Moreover, the *AtTCTP2* mRNA detection is in agreement with previous work showing that the expression pattern of the promoter region of *AtTCTP2* is active in the root with an atypical pattern in the lateral root primordia, similar to the *PUCHI* gene (Toscano-Morales et al., submitted; Karim et al., 2009).

**Figure 6:**
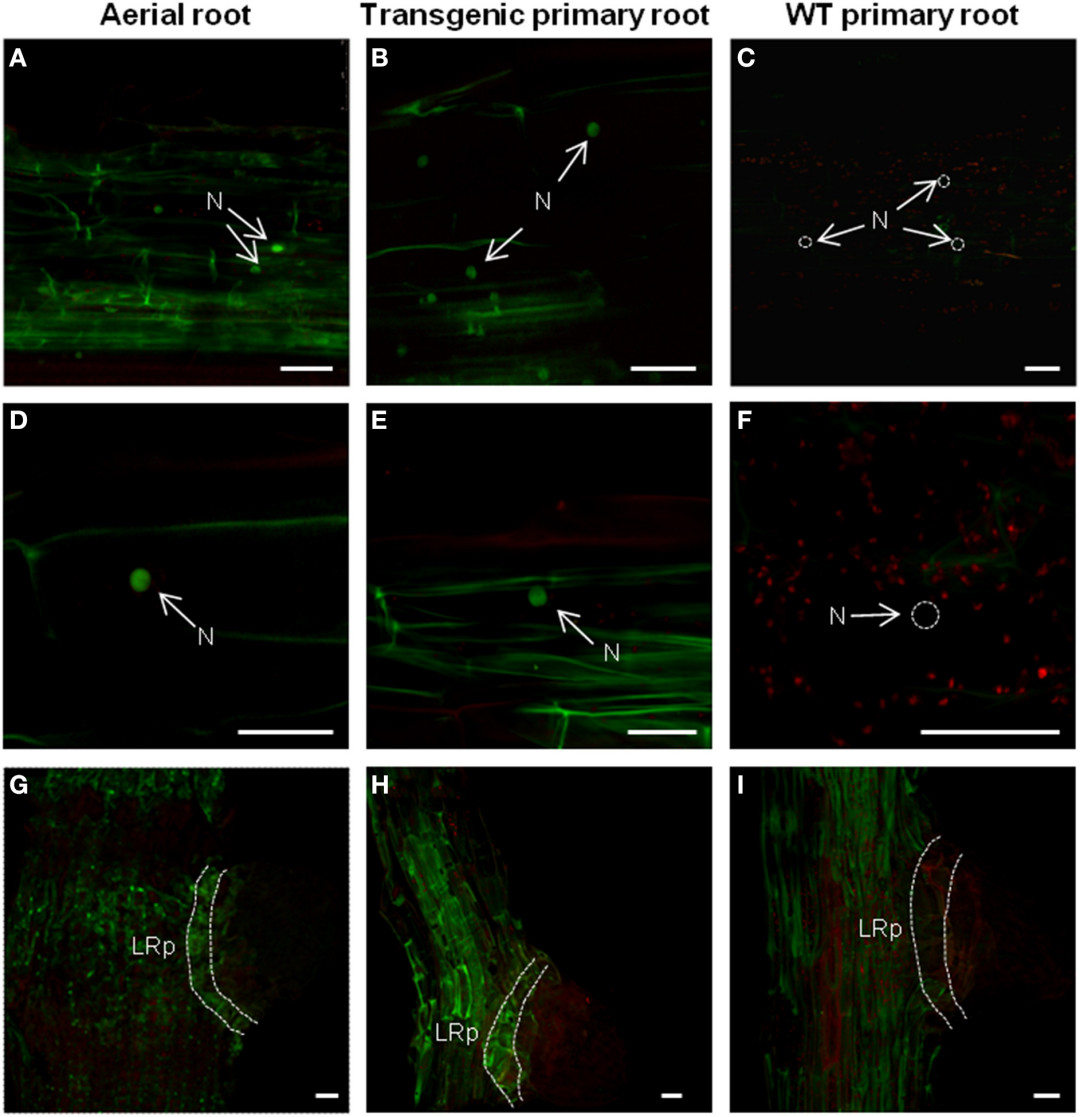
**Comparison of the protein localization pattern between adventitious and primary roots in heterografts**. Mid-root region from aerial or primary roots were used to identify AtTCTP2-GFP signal through fluorescence confocal microscopy. AtTCTP2-GFP nuclear localization (N) in the mid-root region of **(A,D)** aerial and **(B,E)** transgenic primary roots was recurrent in all cases, while in the **(C,F)** WT primary control no nuclear signal was found (N; dashed white circles). In addition, AtTCTP2-GFP was located in lateral root primordia (LRp) both in **(G)** aerial roots and **(H)** transgenic primary roots in contrast to **(I)** WT primary roots. Size bars: 50 μm.

In addition, confocal fluorescence laser microscopy was performed to detect AtTCTP2-GFP protein signal and determine its localization as well. The AtTCTP2-GFP fluorescent signal was specifically found in the nuclei of the mid-root region in all aerial roots that emerged (Figure 6A) which is consistent with the localization pattern found in the *35S::AtTCTP2-GFP* transgenic primary roots (Figure 6B; Toscano-Morales et al., submitted) and in contrast with the absence of nuclear signal in the primary mid-root region of WT stock controls (Figure 6C). Interestingly, no nuclear localization signal is predicted for AtTCTP2, or several plant TCTPs tested, using a nuclear predictor server (http://www.sbc.su.se/~maccallr/nucpred/). In a similar manner, the same localization pattern was observed in lateral root primordia both in aerial and transgenic primary root (Figures 6D,E,G,H), unlike the WT stock control (Figures 6F,I). Remarkably, the protein accumulation pattern (in cells at the base of the lateral root primordia) observed in all aerial roots of WT/*35S::AtTCTP2-GFP* scions was reminiscent of the transgenic primary roots, suggesting an important role of AtTCTP2 and its nuclear localization in midroot growth and lateral root development; this also suggests that AtTCTP2 is capable of moving into the scion and into nuclei of aerial roots, which appear to be induced by AtTCTP2 itself. It must be noted that in these roots fluorescence is not restricted to the nucleus. Instead, signal can also be observed in the cell periphery, and, at lower levels, in cytoplasm; this is much higher than the background autofluorescence of WT roots.

## Discussion

In general, several plant TCTPs appear to move long distance as mRNA or protein by interaction with a phloem RNA-binding protein (Aoki et al., 2005). For instance, the pumpkin *CmTCTP* mRNA has been found circulating in phloem sap exudates and localized to mature phloem (Hinojosa-Moya et al., 2013); also, the *Ricinus communis* and *Lupinus albus TCTP* mRNAs have been found in phloem sap exudate transcriptomes (Doering-Saad et al., 2006; Rodríguez-Medina et al., 2011). Likewise, CmTCTP has been found in the pumpkin phloem sap exudate proteome (Lin et al., 2009). However, the capacity of either TCTP protein or mRNA to move across a graft union had not been obtained to date.

Detection of both *AtTCTP2-GFP* mRNA and protein in distant sites from the graft strongly indicates the capacity of AtTCTP2 to move long distance and suggests its probable role as a non-cell autonomous protein and/or RNA. Regarding the movement of the mRNA, the fact that the ORF was detected in WT scions indicates that this does not require either the 5′ or 3′UTR of this transcript; also, that this mRNA is capable of transporting heterologous mRNAs (*GFP* mRNA, in the present case). The strategy used in the present study (i.e., fusing the *AtTCTP2* and *GFP* ORFs and detection of the former with oligonucleotide primers directed against the latter) ensured that only the transgene mRNA would be detected if it were capable of moving into WT scion or stock. Tobacco harbors two *TCTP* genes; these were not amplified using primers directed against *AtTCTP2* (Figure S1; not shown).

Moreover, the emergence of aerial adventitious roots in grafts where the protein was detected, but not the transcript, together with its observed localization pattern, suggest a direct correlation between the presence of AtTCTP2 and development of aerial adventitious roots, as well as supporting the notion that this protein is transported through a graft union and thus long-distance. The latter may indicate a probable role of AtTCTP2 in cell reprogramming, as has been suggested for other TCTPs (Gutiérrez-Galeano et al., 2014). Additionally, the presence of *AtTCTP2-GFP* mRNA in all adventitious and transgenic primary roots suggests that this gene could be transcriptionally or posttranscriptionally regulated, probably in response to stress signals such as changes in hormonal and nutritional levels. That the endogenous TCTP genes are probably not involved is suggested by the fact that their mRNA levels were not altered due to the grafting procedure or the heterologous expression of AtTCTP-GFP (Figure S2).

Our results indicate that AtTCTP2 localizes to root cortex nuclei, in both the rootstock and grafted scions. Interestingly, this protein probably lacks a nuclear localization signal. Thus, it is possible that this protein is chaperoned by other protein(s) to the nucleus, or, because of its relatively small size it may be able to diffuse into it. It has been suggested that lateral root development and plant regeneration are related; given the localization of AtTCTP2 in lateral root primordia, this protein could be one of the links between both phenomena (Sugimoto et al., 2010).

Adventitious roots arise from the pericycle, from which all cell types form *de novo* (Atkinson et al., 2014; Bellini et al., 2014). Several types of stress, such as mechanical damage and hypoxia, induce these roots and thus have an adaptive value. As with lateral roots, auxin signaling is involved in the formation of adventitious roots, while its positioning is regulated by ethylene (Bellini et al., 2014). In addition, some plant species, such as potato (*Solanum tuberosum*), depend on aerial roots or stems for propagation through plantlet nodes (stolons), which are important mechanisms for vegetative reproduction in several plant species (Bellini et al., 2014). Interestingly, AtTCTP2 is structurally related to the potato TCTP (Gutiérrez-Galeano et al., 2014) suggesting a role of AtTCTP2 and the structurally related CmTCTP in controlling cell differentiation in roots to induce plant regeneration (Hinojosa-Moya et al., 2013; Toscano-Morales et al., submitted). The role of this protein in shoot tissues is less clear.

Our results strongly suggest that AtTCTP2 protein and mRNA have supracellular functions, based in their capacity to move to distant tissues, likely through the phloem. However, several questions arise: does the AtTCTP2 long distance movement (as protein or transcript) is linked to its function? Do all other plant TCTPs from vascular plants share the capacity for long distance movement, or is it a specific feature of some TCTPs? Clearly, more experimental work is needed to address these important issues. However, this work provides a foundation for the analysis of long-distance movement of plant TCTPs and the function of such movement.

## Concluding remarks

The specific mechanism for long-distance movement of AtTCTP2 is still unclear. Furthermore, if this mRNA or protein movement is related to its function is yet to be resolved. The fact that AtTCTP2 was able to move long distance in tobacco grafts either as mRNA or protein was strongly suggested from the RT-PCR and fluorescence confocal microscopy results. Moreover, the aerial root emergence was related only to the protein long-distance movement reinforcing the notion that AtTCTP2 is related to cell reprogramming.

## Author contributions

Roberto Toscano-Morales performed most experiments; Angélica C. Martínez-Navarro carried out the Western Blot experiments; Roberto Ruiz-Medrano, Roberto Toscano-Morales, and Beatriz Xoconostle-Cázares devised the experimental plan. Roberto Toscano-Morales and Roberto Ruiz-Medrano wrote the manuscript. Beatriz Xoconostle-Cázares and Roberto Ruiz-Medrano obtained financial support for the work. All authors read and approved the final version of the manuscript.

### Conflict of interest statement

The authors declare that the research was conducted in the absence of any commercial or financial relationships that could be construed as a potential conflict of interest.

## Supplementary material

Figure S1 **AtTCTP2 and NtTCTP nucleotide and amino acid alignments**. Divergent nucleotide and amino acid regions between these two sequences are shown (**A** and **B**, respectively).

Figure S2 **Comparison of NtTCTP transcript levels between transgenic and WT stocks or scions**. Using the same total RNA extracted previously from stocks or scions, samples were homogenized to a given concentration (100 ng/μl) to be used as templates. **(A)** Specific primers were used to detect a unique sequence for NtTCTP ORF that does not amplify any region within the AtTCTP2 ORF to detect the levels of NtTCTP mRNA. **(B)** qRT PCR was performed and arbitrary fluorescence units detected for NtTCTP in transgenic (AtTCTP2-GFP) stocks or scions (blue bars) were compared to their WT counterparts (green bars) in grafts after being normalized against 18S as reference. At least three biological and technical replicates were performed in each case, given as means ± SE.

## References

[B1] Aoki K. Suzui N. Fujimaki S. Dohmae N. Yonekura-Sakakibara K. Fujiwara T.. (2005). Destination-selective long-distance movement of phloem proteins. Plant Cell 17, 1801–1814. 10.1105/tpc.105 15863519 PMC1143078

[B2] Atkinson J. A. Rasmussen A. Traini R. Voss U. Sturrock C. J. Mooney S. J.. (2014). Branching out in roots: uncovering form, function and regulation. Plant Physiol. 166, 538–550. 10.1104/pp.114.245423 25136060 PMC4213086

[B4] Bellini C. Pacurar D. I. Perrone I. (2014). Adventitious roots and lateral roots: similarities and differences. Annu. Rev. Plant Biol. 65, 639–666. 10.1146/annurev-arplant-050213-035645 24555710

[B5] Berkowitz O. Jost R. Pollmann S. Masle J. (2008). Characterization of TCTP, the translationally controlled tumor protein, from *Arabidopsis thaliana*. Plant Cell 20, 3430–3447. 10.1105/tpc.108.061010 19060111 PMC2630444

[B6] Bommer U. A. (2012). Cellular function and regulation of the Translationally Controlled Tumour Protein TCTP. Open Allergy J. 5, 19–32 10.2174/1874838401205010019

[B7] Bommer U. A. Thiele B. J. (2004). The translationally controlled tumour protein (TCTP). Int. J. Biochem. Cell Biol. 36, 379–385. 10.1016/S1357-2725(03)00213-9 14687915

[B8] Brioudes F. Thierry A. M. Chambrier P. Mollereau B. Bendahmane M. (2010). Translationally controlled tumor protein is a conserved mitotic growth integrator in animals and plants. Proc. Natl. Acad. Sci. U.S.A. 107, 16384–16389. 10.1073/pnas.1007926107 20736351 PMC2941279

[B9] Cans C. Passer B. J. Shalak V. Nancy-Portebois V. Crible V. Amzallag N.. (2003). Translationally controlled tumor protein acts as a guanine nucleotide dissociation inhibitor on the translation elongation factor eEF1A. Proc. Natl. Acad. Sci. U.S.A. 100, 13892–13897. 10.1073/pnas.2335950100 14623968 PMC283517

[B10] Cao B. Lu Y. Chen G. Lei J. (2010). Functional characterization of the translationally controlled tumor protein (TCTP) gene associated with growth and defense response in cabbage. Plant Cell Tissue Organ Cult. 103, 217–226 10.1007/s11240-010-9769-6

[B11] Chen S. H. Wu P. S. Chou C. H. Yan Y. T. Liu H. Weng S. Y.. (2007). A knockout mouse approach reveals that TCTP functions as an essential factor for cell proliferation and survival in a tissue- or cell type-specific manner. Mol. Biol. Cell 18, 2525–2532 10.1091/mbc.E07-02-0188 17475776 PMC1924818

[B12] de Souza C. R. Carvalho L. J. de Mattos C. J. (2004). Comparative gene expression study to identify genes possibly related to storage root formation in cassava. Protein Pept. Lett. 11, 577–582. 10.2174/0929866043406319 15579128

[B13] Doering-Saad C. Newbury H. J. Couldridge C. E. Bale J. S. Pritchard J. (2006). A phloem-enriched cDNA library from Ricinus: insights into phloem function. J. Exp. Bot. 57, 3183–3193. 10.1093/jxb/erl082 16936221

[B14] Drew M. Jackson M. Giffard S. (1979). Ethylene-promoted adventitious rooting and development of cortical air spaces (aerenchyma) in roots may be adaptive responses to flooding in *Zea mays* L. Planta 147, 83–88. 10.1007/BF00384595 24310899

[B15] Gutiérrez-Galeano D. F. Toscano-Morales R. Calderón-Pérez B. Xoconostle-Cázares B. Ruiz-Medrano R. (2014). Structural divergence of plant TCTPs. Front. Plant Sci. 5:361. 10.3389/fpls.2014.00361 25120549 PMC4114181

[B18] Hinojosa-Moya J. Xoconostle-Cázares B. Toscano-Morales R. Ramírez-Ortega F. Cabrera-Ponce J. L. Ruiz-Medrano R. (2013). Characterization of the pumpkin Translationally-Controlled Tumor Protein CmTCTP. Plant Signal. Behav. 8:e26477. 10.4161/psb.26477 24065051 PMC4091340

[B19] Hsu Y. C. Chern J. J. Cai Y. Liu M. Choi K. W. (2007). *Drosophila* TCTP is essential for growth and proliferation through regulation of dRheb GTPase. Nature 445, 785–788. 10.1038/nature05528 17301792

[B20] Imlau A. Truernit E. Sauer N. (1999). Cell-to-cell and long-distance trafficking of the green fluorescent protein in the phloem and symplastic unloading of the protein into sink tissues. Plant Cell 11, 309–322. 10.1105/tpc.11.3.309 10072393 PMC144181

[B21] Jones A. M. Thomas V. Bennett M. H. Mansfield J. Grant M. (2006). Modifications to the Arabidopsis defense proteome occur prior to significant transcriptional change in response to inoculation with *Pseudomonas syringae*. Plant Physiol. 142, 1603–1620. 10.1104/pp.106.086231 17028151 PMC1676056

[B23] Karim M. R. Hirota A. Kwiatkowska D. Tasaka M. Aida M. (2009). A role for Arabidopsis PUCHI in floral meristem identity and bract suppression. Plant Cell 21:1360–1372. 10.1105/tpc.109.067025 19482972 PMC2700531

[B24] Kim G. LeBlanc M. L. Wafula E. K. dePamphilis C. W. Westwood J. H. (2014). Plant science. Genomic-scale exchange of mRNA between a parasitic plant and its hosts. Science 345, 808–811. 10.1126/science.1253122 25124438

[B27] Kim Y. M. Han Y. J. Hwang O. J. Lee S. S. Shin A. Y. Kim S. Y.. (2012). Overexpression of Arabidopsis translationally controlled tumor protein gene AtTCTP enhances drought tolerance with rapid ABA-induced stomatal closure. Mol. Cells 33, 617–626. 10.1007/s10059-012-0080-8 22610367 PMC3887759

[B28] Lin M. K. Lee Y. J. Lough T. J. Phinney B. S. Lucas W. J. (2009). Analysis of the pumpkin phloem proteome provides insights into angiosperm sieve tube function. Mol. Cell Proteomics 8, 343–356. 10.1074/mcp.M800420-MCP200 18936055

[B45] Livak K. J. Schmittgen T. D. (2001). Analysis of relative gene expression data using real-time quantitative PCR and the 2^−ΔΔ*C*_*T*_^ Method. Methods 25, 402–408 10.1006/meth.2001.1262 11846609

[B30] Lopez A. R. Franco A. P. (2006). Cloning and expression of cDNA encoding translationally controlled tumor protein from strawberry fruits. Biol. Plant. 50, 447–449 10.1007/s10535-006-0067-4

[B32] Naiman R. J. Décamps H. (1997). The ecology of interfaces: riparian zones. Annu. Rev. Ecol. Evol. Syst. 28, 621–658 10.1146/annurev.ecolsys.28.1.621

[B33] Nakkaew A. Chotigeat W. Phongdara A. (2010). Molecular cloning and expression of EgTCTP, encoding a calcium binding protein, enhances the growth of callus in oil palm (*Elaeis guineensis*, Jacq). Songklanakarin J. Sci. Technol. 562, 561–569.

[B34] Palauqui J. C. Elmayan T. Pollien J. M. Vaucheret H. (1997). Systemic acquired silencing: transgene-specific post-transcriptional silencing is transmitted by grafting from silenced stocks to non-silenced scions. EMBO J. 16, 4738–4745. 10.1093/emboj/16.15.4738 9303318 PMC1170100

[B35] Qin X. Gao F. Zhang J. Gao J. Lin S. Wang Y. (2011). Embryo formation regulation of the endosperm development molecular cloning, characterization and expression of cDNA encoding translationally controlled tumor protein (TCTP) from *Jatropha curcas* L. Mol. Biol. Rep. 38, 3107–3112 10.1007/s11033-010-9980-x 20140648

[B36] Rodríguez-Medina C. Atkins C. A. Mann A. J. Jordan M. E. Smith P. M. C. (2011). Macromolecular composition of phloem exudate from white lupin (*Lupinus albus* L.). BMC Plant Biol. 11:36. 10.1186/1471-2229-11-36 21342527 PMC3055823

[B38] Sage-Ono K. Ono M. Harada H. Kamada H. (1998). Dark-induced accumulation of mRNA for a homolog of translationally controlled tumor protein (TCTP) in Pharbitis. Plant Cell Physiol. 39, 357–360. 10.1093/oxfordjournals.pcp.a029377 9588028

[B39] Sugimoto K. Jiao Y. Meyerowitz E. M. (2010). Arabidopsis regeneration from multiple tissues occurs via a root development pathway. Dev. Cell. 18, 463–471. 10.1016/j.devcel.2010.02.004 20230752

[B40] Thaw P. Baxter N. J. Hounslow A. M. Price C. Waltho J. P. Craven C. J. (2001). Structure of TCTP reveals unexpected relationship with guanine nucleotide-free chaperones. Nat. Struct. Biol. 8, 701–704. 10.1038/90415 11473261

[B41] Wang X. Fonseca B. D. Tang H. Liu R. Elia A. Clemens M. J.. (2008). Re-evaluating the roles of proposed modulators of mammalian target of rapamycin complex 1 (mTORC1) signaling. J. Biol. Chem. 283, 30482–30492. 10.1074/jbc.M803348200 18676370 PMC2662142

[B42] Woo H. H. Hawes M. C. (1997). Cloning of genes whose expression is correlated with mitosis and localized in dividing cells in root caps of *Pisum sativum* L. Plant Mol. Biol. 35, 1045–1051. 10.1023/A:1005930625920 9426627

[B46] Xoconostle-Cázares B. Xiang Y. Ruiz-Medrano R. Hong-Li W. Monzer J. Byung-Chun Y.. (1999). Plant paralog to viral movement protein that potentiates transport of mRNA into the phloem. Science 283, 94–98. 9872750 10.1126/science.283.5398.94

[B43] Yan A. H. Zhang L. F. Zhang Y. W. Wang D. M. (2009). Early stage SSH library construction of wheat near-isogenic line TcLr19 under the stress of *Puccinia recondita* f. sp. Tritici. Front. Agric. China 3:146–151 10.1007/s11703-009-0045-7

