# Supplementary figures and images for: Long distance movement of an Arabidopsis Translationally Controlled Tumor Protein (AtTCTP2) mRNA and protein in tobacco
Source: Front Plant Sci. 2014 Dec 17;5:705. doi: 10.3389/fpls.2014.00705 (PMC4269120; doi:10.3389/fpls.2014.00705)

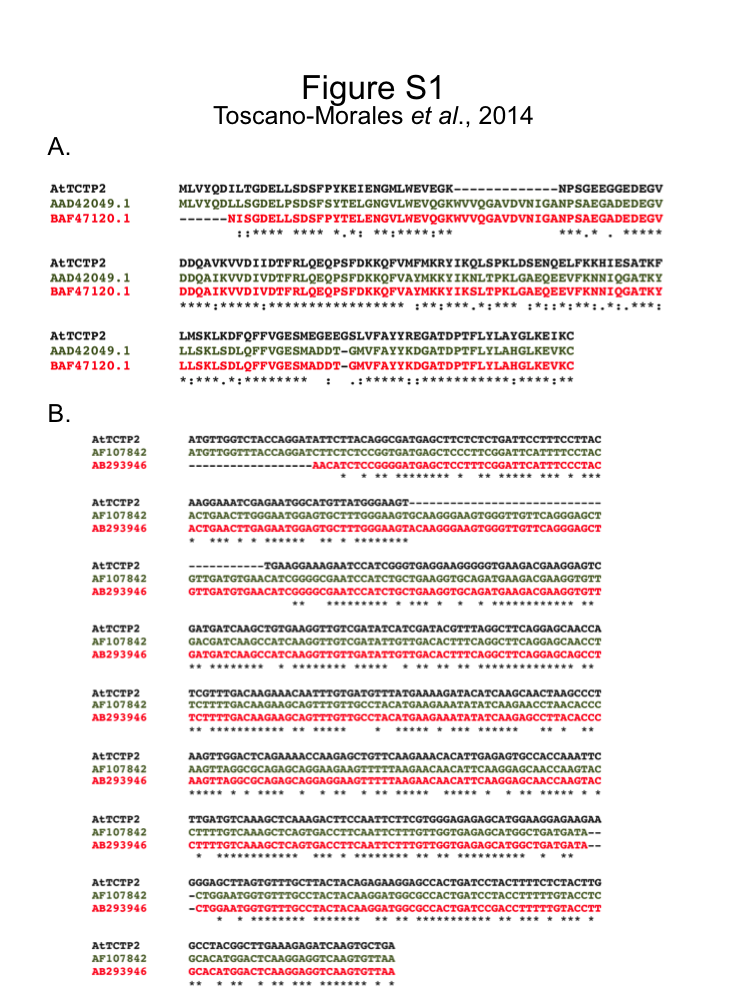

Supplement: Figure S1 — AtTCTP2 and NtTCTP nucleotide and amino acid alignments. Divergent nucleotide and amino acid regions between these two sequences are shown (A and B, respectively). [file Image1.TIF]

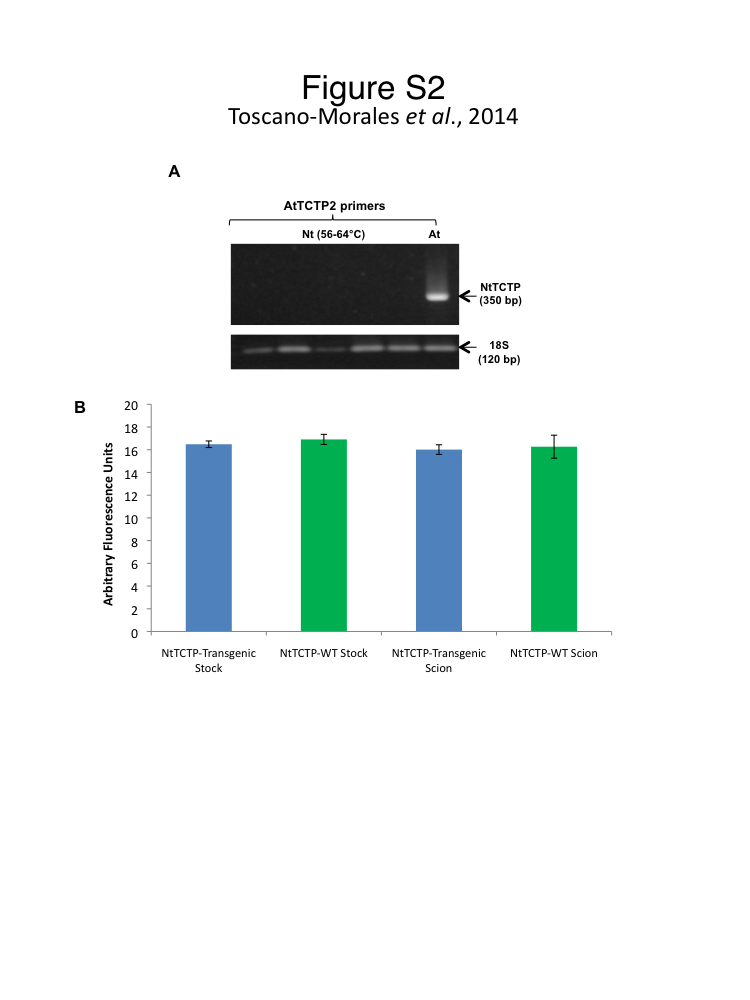

Supplement: Figure S2 — Comparison of NtTCTP transcript levels between transgenic and WT stocks or scions. Using the same total RNA extracted previously from stocks or scions, samples were homogenized to a given concentration (100 ng/μl) to be used as templates. (A) Specific primers were used to detect a unique sequence for NtTCTP ORF that does not amplify any region within the AtTCTP2 ORF to detect the levels of NtTCTP mRNA. (B) qRT PCR was performed and arbitrary fluorescence units detected for NtTCTP in transgenic (AtTCTP2-GFP) stocks or scions (blue bars) were compared to their WT counterparts (green bars) in grafts after being normalized against 18S as reference. At least three biological and technical replicates were performed in each case, given as means ± SE. [file Image2.TIF]
